# Comparing McGRATH® MAC, C-MAC®, and Macintosh Laryngoscopes Operated by Medical Students: A Randomized, Crossover, Manikin Study

**DOI:** 10.1155/2016/8943931

**Published:** 2016-09-15

**Authors:** Myungju Shin, Sun Joon Bai, Ki-Young Lee, Ein Oh, Hyun Joo Kim

**Affiliations:** Department of Anesthesiology and Pain Medicine and the Anesthesia and Pain Research Institute, Yonsei University College of Medicine, 50-1 Yonsei-ro, Seodaemun-gu, Seoul 120-752, Republic of Korea

## Abstract

We hypothesized that the McGRATH MAC would decrease the time of intubation compared to C-MAC for novices. Thirty-nine medical students who had used the Macintosh blade to intubate a manikin fewer than 3 times were recruited. The participants performed sequential intubations on the manikin in two simulated settings that included a normal airway and a difficult airway (tongue edema). The intubation time, success rate of intubation, Cormack-Lehane grade at laryngoscopy, and difficulty using the device were recorded. Each participant was asked to identify the device that was most useful. The intubation time decreased significantly and by a similar amount to the McGRATH MAC and C-MAC compared to the Macintosh blade (*P* < 0.001 and *P* = 0.017, resp.). In the difficult airway, the intubation times were similar among the three devices. The McGRATH MAC and C-MAC significantly increased the success rate of intubation, improved the Cormack-Lehane grade, and decreased the difficulty score compared to the Macintosh blade in both airway settings. The majority of participants selected the McGRATH MAC as the most useful device. The McGRATH MAC and C-MAC may offer similar benefits for intubation compared to the Macintosh blade in normal and difficult airway situations.

## 1. Introduction

Tracheal intubation is critical for securing the airway in various situations. The American Society of Anesthesiologists' Closed Claim study showed that difficult intubation or esophageal intubation is the cause of the approximately 35% of life-threatening respiratory events, including death and permanent brain damage [1]. However, the skill needed for a successful direct laryngoscopic tracheal intubation is complex and difficult to acquire because the individual performing the procedure needs to align the oral, pharyngeal, and tracheal axes to obtain a view of the glottis [2, 3]. Moreover, an emergent tracheal intubation in the prehospital setting or an airway of unanticipated difficulty can further complicate the procedure and decrease the success rate [2]. Even when a physician is skilled in the technique, it is difficult to maintain proficiency due to limited clinical opportunities, particularly for nonanesthetists, which results in increased morbidity and mortality [4, 5]. Therefore, an intubating device that can decrease the intubation time and increase the success rate, particularly for medical students and paramedics who have fewer training opportunities, should be an ongoing focus of research and development.

The video laryngoscope has recently become popular in operating rooms and emergency departments. Moreover, the importance of the video laryngoscope has been acknowledged, and this instrument has been incorporated into difficult airway management guidelines [6, 7]. The primary advantage of the video laryngoscope is that the camera on the tip of the blade makes overlapping the laryngeal, pharyngeal, and oral axes unnecessary and the intubation time faster [8, 9]. Among many types of video laryngoscopes available, the C-MAC PM video laryngoscope (Karl Storz, Tuttlingen, Germany) has been verified by multiple clinical studies as appropriate for use both in and outside of hospitals [9–11]. C-MAC has been associated with a shorter intubation time than the Macintosh blade [9, 12]. C-MAC's blade angle is regular and similar to that of the Macintosh blade, and its LCD screen rests on the handle, making the device portable.

**Figure 1:**
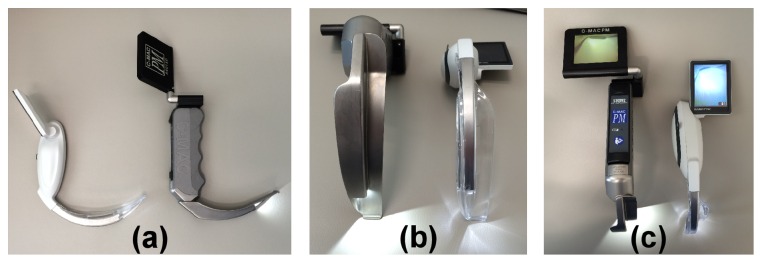
The McGRATH MAC and C-MAC. (a) Lateral view: left, McGRATH MAC, and right, C-MAC. (b) Blade design: left, C-MAC, and right, McGRATH MAC. (c) Anterior view: left, C-MAC, and right, McGRATH MAC.

McGRATH MAC (Aircraft Medical Ltd., Edinburgh, UK) video laryngoscope was recently developed [13, 14], and it has a slim, disposable, transparent, regularly shaped blade similar to the Macintosh blade and a wide LCD screen attached to the handle. Although the McGRATH MAC and C-MAC have the same blade angle and portability, the McGRATH MAC differs from C-MAC in that it is lighter and has a more compact screen and handle and a slimmer blade, and its screen has greater proximity to the axis of the blade (Figure 1). These characteristics of the McGRATH MAC may make tracheal intubation easier and faster, particularly for novices such as medical students, than when using C-MAC. To the best of our knowledge, no study has compared the utility of C-MAC and McGRATH MAC for medical students.

Therefore, the aim of this study was to compare the efficacy of the McGRATH MAC and C-MAC for use by medical students. We hypothesized that the McGRATH MAC would decrease the time of intubation compared to C-MAC in both normal and difficult airway situations due to its more practical LCD screen position, slimmer blade, and lower weight. To investigate this hypothesis, we compared the time that medical students required for intubation of a manikin using the Macintosh blade, the McGRATH MAC, and C-MAC.

## 2. Methods

### 2.1. Study Design and Recruitment

This randomized, crossover study was approved by the Institutional Review Board of Severance Hospital (ref: 1-2015-0020) in Seoul, Republic of Korea, and the need to obtain written informed consent was waived. The study was registered at ClinicalTrials.gov (ref: NCT02458534, May 26, 2015). Thirty-nine medical students (30 males and 9 females) who had performed fewer than 3 intubations on manikins using the Macintosh blade were recruited. This study was conducted at the Clinical Simulation Center at Yonsei University College of Medicine between May and July 2015. Our hospital offers an elective training course on intubation for the medical students, and this study was conducted during that course.

### 2.2. Equipment

A SimMan manikin (Laerdal Medical Canada Ltd., Toronto, ON, Canada) was used. Three devices, including C-MAC PM (Karl Storz, Tuttlingen, Germany), the McGRATH MAC (Aircraft Medical Ltd., Edinburgh, UK), and the Macintosh laryngoscope, were used for intubation. A blade size of 3 was used for all devices. We used a 7.5 mm cuffed endotracheal tube (Mallinckrodt™ TaperGuard Oral/Nasal Tracheal tube, Covidien, MA, USA) for every intubation. A malleable plastic stylet (Portex™ intubation stylet, Smiths Medical ASD, Inc., Norwell, MA, USA) bent with a hockey stick curvature was used for all devices [13, 15].

### 2.3. Experiment

All participants watched a video demonstration of intubation techniques for the three devices and were provided with an additional 15-minute oral explanation on how to use the intubation devices and how to determine the Cormack-Lehane laryngoscope grade [16]. After the instruction session, the participants were allowed to perform one intubation with each device with a normal airway. The participants then performed sequential intubations on the manikin in two different simulated situations, including a normal airway and a difficult airway. Participants received a 30-minute break between the airway settings. The tongue edema was set to maximum level to simulate a difficult airway of Mallampati grade 3 or 4, which is known as the risk level for a difficult tracheal intubation [7, 17] and is commonly encountered in clinical practice [9]. Each participant was allowed to perform up to three intubation attempts with each device in each simulated airway setting. The order of use for the devices was randomized using six allocation sequences developed before the start of the study. Therefore, each participant was allowed to perform a total of eighteen intubation attempts during the study. Participants were required to grade the laryngeal view according to the Cormack-Lehane classification in each intubation attempt [16].

### 2.4. Data Collection

The primary outcome was intubation time, which was defined as the time between when the tip of the blade passed the manikin's teeth and when the first chest expansion was observed using the resuscitation bag after removing the intubating stylet. The secondary outcomes were the number of intubation attempts, the success rate of intubation, the Cormack-Lehane grade at laryngoscopy, and the difficulty of using the device. An intubation attempt was halted if the tip of the blade came out of the mouth. A failed attempt was defined as either an intubation that took longer than 120 seconds or the insertion of the tube into the esophagus [18–20]. A failure to intubation was determined if the participant had not succeeded after three attempts [20, 21]. The difficulty of using the device was recorded on a scale of 0 (extremely easy) to 10 (extremely difficult) after the completion of the intubations using three devices in either the normal airway or difficult airway. Each participant was also asked which device was the easiest to use and why they chose that device.

### 2.5. Statistical Analysis

This study had a crossover design. Sample size was calculated using *α* = 0.05 and *β* = 0.2. A minimum of 4 participants was required in each randomized sequence to detect a 15-second difference [22] with a standard deviation of 18 seconds, which was based on a previous study [13]. Therefore, we estimated that a total of 24 participants would be required. We enrolled 39 participants in the study to minimize data loss.

We compared the primary outcome among the three devices using a linear mixed model (LMM). For the secondary outcomes, such as the success rate of intubation, the number of intubation attempts, the Cormack-Lehane grade, and the difficulty score, an LMM was also used. The results were analyzed using SAS version 9.2 (SAS Institute Inc., Cary, NC, USA.). Data are presented as the median (interquartile range) or number (percentage). A *P* value less than 0.05 was considered statistically significant.

## 3. Results

Thirty-nine medical students participated in this study. All participants performed nine intubation attempts in each airway setting, and no data were excluded. As a result, a total of 702 intubation attempts were performed in the two airway settings.

**Figure 2:**
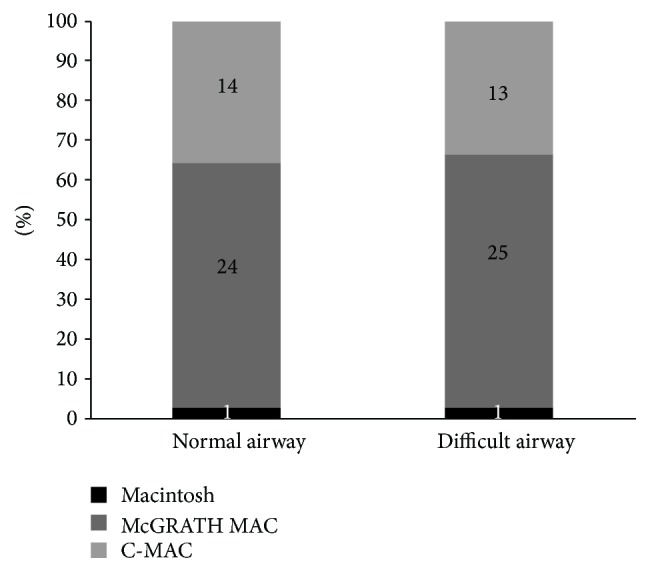
Assessment of the most useful equipment.

In the normal airway, one participant failed to intubate the manikin after three attempts using the Macintosh blade, while all intubations were successful after three attempts with the McGRATH MAC and C-MAC (Table 1). The intubation time on the first attempt did not differ significantly among the three devices in the normal airway. However, the intubation times on the second and third attempts decreased significantly with the McGRATH MAC and C-MAC compared to the Macintosh blade in the normal airway (*P* = 0.005 and *P* = 0.037 on the second attempt and *P* < 0.001 and *P* = 0.001 on the third attempt, resp.). The overall mean intubation time of the three attempts decreased significantly with the McGRATH MAC and C-MAC compared to the Macintosh blade in the normal airway (*P* < 0.001 and *P* = 0.017, resp.). The success rate of intubation on the first, second, and third attempts increased significantly with the McGRATH MAC and C-MAC compared to the Macintosh blade in the normal airway (*P* < 0.001 on the first attempt, *P* < 0.001 on the second attempt, and *P* = 0.012 on the third attempt). The success rate of intubation after three attempts did not differ significantly among the three devices. The number of attempts decreased significantly with the McGRATH MAC and C-MAC compared to the Macintosh blade (*P* = 0.002). The Cormack-Lehane grade improved significantly on the first, second, and third attempts with the McGRATH MAC and C-MAC compared to the Macintosh blade (*P* < 0.001). The difficulty score of using the device was higher for the Macintosh blade compared to the McGRATH MAC and C-MAC (*P* = 0.002). No parameters, including intubation time, success rate of intubation, number of attempts, Cormack-Lehane grade, and difficulty score, differed significantly between the McGRATH MAC and C-MAC. Twenty-four participants chose the McGRATH MAC as the most useful device in a normal airway, while fourteen participants chose C-MAC, and one chose the Macintosh blade (Figure 2).

In the difficult airway (tongue edema), 12 participants failed to intubate the manikin with the Macintosh blade, while one failed with the McGRATH MAC, and two failed with C-MAC (Table 2). The intubation time on the three attempts did not differ significantly among the three devices. The success rate of intubation on the first, second, and third attempts increased significantly with the McGRATH MAC and C-MAC compared to the Macintosh blade (*P* < 0.001). The overall success rate increased significantly with the McGRATH MAC and C-MAC compared to the Macintosh blade (*P* < 0.001). The Cormack-Lehane grade improved significantly with the McGRATH MAC and C-MAC compared to the Macintosh blade (*P* < 0.001). The difficulty score of using the device was significantly higher for the Macintosh blade compared to the McGRATH MAC and C-MAC (*P* < 0.001). No parameters, including intubation time, success rate of intubation, number of attempts, Cormack-Lehane grade, and difficulty score, differed significantly between the McGRATH MAC and C-MAC. Twenty-five participants chose the McGRATH MAC as the most useful device in the difficult airway, while thirteen participants chose C-MAC, and one chose the Macintosh blade (Figure 2).

Participants chose the McGRATH MAC as the most useful device for intubation because of its lighter weight (53%), comfortable grip (22%), smooth performance during the insertion of the device (12%), close proximity of the camera to the blade (6%), ease of moving the tongue anteriorly with the blade (4%), and simplicity and compact size (2%). Participants selected C-MAC as the most useful device for intubation because of its stability of the blade which eliminates shaking (70%) and its comfortable grip (30%).

## 4. Discussion

In the manikin with the normal airway, the intubation time decreased significantly with the McGRATH MAC and C-MAC compared to the Macintosh blade, although there was no significant difference between the McGRATH MAC and C-MAC. In the difficult airway caused by tongue edema, the intubation time did not differ significantly among the three devices. The success rate of intubation, the Cormack-Lehane grade, and the difficulty score all improved significantly with the McGRATH MAC and C-MAC compared to the Macintosh blade in both airway scenarios, while there were no significant differences between the McGRATH MAC and C-MAC except in the selection of the McGRATH MAC as the most useful device.

In the normal airway, both video laryngoscopes (the McGRATH MAC and C-MAC) significantly reduced the intubation time compared to the Macintosh blade, although there was no significant difference in the intubation time on the first attempt at intubation. This result agrees with a previous manikin study that involved paramedics and demonstrated that the McGRATH MAC significantly decreased the tracheal intubation time compared to the Macintosh blade in a normal airway [23]. The intubation times were similar between the McGRATH MAC and Macintosh blade in another manikin study that involved medical students [13] and were also similar between C-MAC and Macintosh blade in yet another manikin study that involved experienced anesthetists. We think that this is because the video laryngoscope is very easy to learn and requires less than six intubation attempts to achieve a success rate of more than 90%, as shown in a previous study [24], while approximately 50 attempts are needed to achieve proficiency with the Macintosh blade [25]. Moreover, the McGRATH MAC and C-MAC improve the laryngeal view compared to the Macintosh blade. This result is comparable with those of previous studies, which demonstrated that the Cormack-Lehane grade improves with the use of a video laryngoscope because the camera on the blade tip eliminates the need to align the oral, pharyngeal, and laryngeal axes [13, 18, 26]. The success rate of intubation increased, the number of intubation attempts decreased, and the difficulty score decreased significantly with the McGRATH MAC and C-MAC compared to the Macintosh blade. Our result is inconsistent with a previous manikin study showing that C-MAC did not influence the success rate of intubation, the number of the intubation attempts, or the difficulty score compared to the Macintosh blade in a normal airway [26]. We think that this difference depends on the target subjects and that study investigated experienced anesthetists with approximately 17 years of experience with the Macintosh blade and did not necessarily apply the video laryngoscope in normal airways. By contrast, in observational or retrospective studies in the emergency department, C-MAC was associated with an improved laryngeal view along with a higher success rate compared to the Macintosh blade, which is consistent with our results [10, 11]. We found no significant differences in the observed parameters between the two types of video laryngoscopes (the McGRATH MAC and C-MAC) except for the identification of the most useful device. Therefore, the McGRATH MAC and C-MAC seem to be similarly beneficial in improving the performance of intubation in a normal airway compared to the Macintosh blade, particularly for medical students.

In the difficult airway, the intubation times were similar among the three devices. Our results agree with a previous manikin study demonstrating that the intubation times were similar between the McGRATH MAC and Macintosh blade in cervical immobilization performed by medical students [13], and this indicates that there may be no benefit to a video laryngoscope on the intubation time in a situation of tongue edema. In another simulated manikin or clinical study of cervical spine immobilization, the intubation time was longer, with a mean of 13 s or similar, when experienced anesthetists used C-MAC compared to the Macintosh blade, and the success rate and laryngeal view were similar between the two devices [27, 28]. By contrast, in our study, the success rate and the laryngeal grade improved significantly with the use of a video laryngoscope, such as C-MAC or the McGRATH MAC, compared to the Macintosh blade. This difference may be due to the lack of familiarity participants had with the Macintosh blade and the more difficult airway situation caused by tongue edema in our study. In another clinical study with patients who were at risk for a difficult intubation, C-MAC provided a higher success rate with an improved laryngeal view [29]. In our study, the difficulty score was significantly lower with C-MAC and McGRATH MAC than with the Macintosh blade. Therefore, based on our study results, it appears that when medical students use the McGRATH MAC or C-MAC, the number of successful tracheal intubations with a difficult airway situation, such as tongue edema, increases, although the intubation times are similar.

To the best of our knowledge, this is the first study to compare the intubation conditions between the McGRATH MAC and C-MAC for novices such as medical students. Several previous studies have investigated the McGrath Series 5 (Aircraft Medical Ltd., Edinburgh, UK), which is the previous version of the McGRATH MAC [30]. The McGrath Series 5 has been shown to increase the success rate of tracheal intubation and to avoid complications, such as esophageal intubation and dental trauma, compared to the Macintosh blade in a normal airway when used by medical students with no intubation experience because of its better laryngeal view [18]. However, the McGrath Series 5 was not reported to reduce intubation time or increase the ease of use compared to the Macintosh blade [18]. With difficult airway simulations, such as cervical spine rigidity, the intubation time was even longer, with a mean of 14 to 80 s, when paramedics or experienced anesthetists used the McGrath Series 5 compared to the Macintosh blade, despite the improvement in the laryngeal view [19, 27, 30]. The McGrath Series 5 does not always guarantee an easy and successful intubation, despite the good laryngeal view [14, 31], which may be because of the disadvantages of the acute angle of the blade, including difficulty with the approach of the tube into the vocal cords even though the vocal cords are easily seen, the advancement of the endotracheal tube into the trachea due to contact with the anterior tracheal wall, and the removal of the stylet from the tube [32, 33]. Moreover, the use of the stylet with the acute angle combined with the endotracheal tube can result in an increased risk of pharyngeal or hypopharyngeal perforation [34–37]. These imperfections have appeared in previous clinical and manikin studies comparing the McGrath Series 5 and C-MAC. In a manikin study of cervical spine immobilization, the success rate was lower, at 28%, and the intubation time was longer, with a mean of 67 s, when experienced anesthetists used the McGrath Series 5 compared to C-MAC [27]. In another clinical study of experienced anesthetists who were very familiar with video laryngoscopes, C-MAC resulted in a faster intubation time, fewer intubation attempts, and a lower difficulty score compared to the McGrath Series 5, despite similar success rates in patients with a Mallampati grade of more than 3 [21]. Therefore, in our study, we did not investigate the video laryngoscope with the acute blade angle but rather investigated the two types of video laryngoscopes with regular-shaped blades that were similar to the Macintosh blade. Our results suggest that a video laryngoscope with a regular-shaped blade can reduce the intubation time and the difficulty of device use even in a normal airway setting and for novices such as medical students.

The McGRATH MAC was chosen as the most useful device by the most of the participants, while the Macintosh blade was selected as the least useful device. This finding is similar to the results of previous studies showing that a video laryngoscope was rated as more useful than the Macintosh blade by novice users [26]. This result may have occurred because the laryngeal grade improves with the use of the video laryngoscope, and medical students can confirm the passage of the tracheal tube through the vocal cords clearly via the images on the screen. In our study, most participants chose the McGRATH MAC as the preferred device rather than C-MAC. We investigated the reasons why the participants preferred the McGRATH MAC and determined that the main reason was its lighter weight. Most participants were satisfied with the lighter weight, the smaller and slimmer blade size, and the handle of the McGRATH MAC, which made them feel they could manipulate the device easily. The closer position of the LCD screen to the blade was also a reason for this preference because it may allow for better eye-hand coordination, resulting in a better performance of tracheal intubation and less strain of the neck muscles, as shown in previous studies [38, 39].

Although the video laryngoscopes are useful, as shown by our results, there are some limitations of the use of these devices. The optimal glottic view does not guarantee a successful intubation [40] because correct positioning of the endotracheal tube toward the vocal cords is required while monitoring the patient's mouth. During this process, the physicians cannot always see the tip of the endotracheal tube, which results in oropharyngeal complications such as palatopharyngeal arch perforation [37]. In addition, all types of airway situations cannot be resolved with the use of video laryngoscopes. A previous study demonstrated that intubation failure occasionally occurred with the GlideScope video laryngoscope especially in patients with altered neck anatomy and the presence of a surgical scar, radiation changes, or a mass [41]. The authors recommended that methods other than the video laryngoscope should be used for patients with neck pathology. Moreover, oropharyngeal secretions or blood can obscure the view on the monitor and make intubation using the video laryngoscope impossible [42]. The benefits of video laryngoscope use can be maximized by understanding these limitations, and further studies on the role of the video laryngoscope may be required.

There are some limitations to our study. First, because the study was performed on manikins, our results may not directly apply to a clinical situation. However, we chose medical students as the target subjects, and, therefore, there was an ethical concern about performing intubations on real patients. In addition, the manikin has been used widely as a validated surrogate in several previous studies that have investigated intubating devices because it allows the intubation environment to be strictly standardized [13, 18, 26, 31]. Second, we did not investigate other difficult intubation conditions, such as cervical immobilization [13, 26, 30], and our results cannot be applied to these situations. We chose the difficult airway setting caused by tongue edema rather than cervical immobilization or pharyngeal obstruction because tongue edema induces the worst laryngeal view, the highest intubation failure rate, and the highest difficulty score of intubation, as shown in a previous study [20], and we think that the utility of the video laryngoscope is most noticeable and important in the most difficult situation. Third, the participants were not blinded to which laryngoscope was being used, and they may have adjusted to the simulated manikin rapidly, making their intubation attempts more successful with the latter devices than with the former [13]. To decrease this bias, the outcomes were clearly defined before the start of the study, and the participants were randomized into 6 allocation sequences. Fourth, a further limitation may be the small number of the participants in this study. However, we calculated the sample size before the start of the study by considering the crossover design. Moreover, the experiences of the participants were consistent, and participants who had experience with any type of video laryngoscope were not recruited to reduce bias in the comparison of the McGRATH MAC and C-MAC.

## 5. Conclusions

The McGRATH MAC and C-MAC resulted in a similar decrease in intubation time compared to the Macintosh blade in the normal airway, while the intubation times were similar among all devices in the difficult airway. The McGRATH MAC and C-MAC resulted in similar improvements in the success rate, laryngeal grade, and difficulty of use compared to the Macintosh blade in both the normal and difficult airways. The McGRATH MAC and C-MAC may have similar benefits in improving intubation conditions in normal and difficult airway situations.

## Figures and Tables

**Table 1:** Intubation time, intubation success rate, Cormack-Lehane grade, and subjective difficulty in the normal airway.

	Macintosh (*n* = 39)	McGRATH® MAC (*n* = 39)	C-MAC® (*n* = 39)	*P* value
Intubation time (s)				
First attempt (s)	26.9 (23.3–30.5)	25.8 (22.5–29.1)	28.5 (25.1–31.8)	0.313
Second attempt (s)	24.7 (22.1–27.3)	20.1 (17.7–22.5)^*∗*^	21.3 (18.9–23.7)^*∗*^	0.017
Third attempt (s)	23.7 (21.7–25.6)	19.5 (17.7–21.3)^†^	19.9 (18.2–21.8)^*∗*^	0.001
Overall attempts (s)	26.6 (24.2–29.1)	21.8 (19.4–24.2)^†^	23.2 (20.8–25.7)^*∗*^	0.003
Success rate of intubation				
First attempt	31 (80)	39 (100)^†^	39 (100)^†^	<0.001
Second attempt	33 (85)	39 (100)^†^	39 (100)^†^	0.002
Third attempt	35 (90)	39 (100)^*∗*^	39 (100)^*∗*^	0.015
Overall attempts	38 (97)	39 (100)	39 (100)	0.373
Number of intubation attempts, 1/2/3	31/5/2	39/0/0^*∗*^	39/0/0^*∗*^	0.001
Cormack-Lehane grade, 1/2/3/4				
First attempt	16/16/2/5	37/2/0/0^†^	36/3/0/0^†^	<0.001
Second attempt	18/15/1/5	37/2/0/0^†^	34/5/0/0^†^	<0.001
Third attempt	18/15/4/2	35/3/1/0^†^	36/2/1/0^†^	<0.001
Subjective difficulty, VAS (0–10)	4.6 (4.1–5.1)	2.2 (1.7–2.7)^†^	2.2 (1.7–2.7)^†^	<0.001

Data are presented as the median (interquartile range) or number (percentage). ^*∗*^ *P* value <0.05 compared to the Macintosh blade. ^†^ *P* value <0.001 compared to the Macintosh blade.

**Table 2:** Intubation time, intubation success rate, Cormack-Lehane grade, and subjective difficulty in the simulated difficult airway (tongue edema).

	Macintosh (*n* = 39)	McGRATH® MAC (*n* = 39)	C-MAC® (*n* = 39)	*P* value
Intubation time (s)				
First attempt (s)	28.9 (23.8–31.1)	30.9 (26.3–35.4)	32.7 (28.2–37.2)	0.311
Second attempt (s)	31.7 (26.1–37.3)	27.6 (23.1–32.2)	30.5 (26.0–34.9)	0.367
Third attempt (s)	30.8 (26.1–35.6)	28.9 (24.8–33.2)	28.2 (23.8–32.5)	0.402
Overall attempts (s)	34.3 (29.3–39.3)	31.7 (27.1–36.3)	32.8 (28.1–37.5)	0.354
Success rate of intubation				
First attempt	25 (64)	35 (90)^†^	36 (92)^†^	<0.001
Second attempt	22 (56)	35 (90)^†^	35 (90)^†^	<0.001
Third attempt	24 (62)	37 (95)^†^	36 (92)^†^	<0.001
Overall attempts	27 (69)	38 (97)^†^	37 (95)^†^	<0.001
Number of intubation attempts, 1/2/3	25/2/0	35/1/2	35/1/1	0.663
Cormack-Lehane grade, 1/2/3/4				
First attempt	2/17/5/15	9/29/1/0^†^	7/30/0/2^†^	<0.001
Second attempt	2/20/4/13	9/28/1/1^†^	6/30/2/1^†^	<0.001
Third attempt	2/20/4/13	11/28/0/0^†^	9/28/0/2^†^	<0.001
Subjective difficulty, VAS (0–10)	7.8 (7.2–8.4)	4.5 (3.9–5.1)^†^	4.8 (4.2–5.4)^†^	<0.001

Data are presented as the median (interquartile range) or number (percentage). ^†^ *P* value <0.001 compared to the Macintosh blade.
